# Validation of the Tampa Scale for Kinesiophobia for Temporomandibular Disorders (TSK-TMD) in patients with painful TMD

**DOI:** 10.1186/s10194-016-0706-x

**Published:** 2016-12-05

**Authors:** Songlin He, Jinhua Wang, Ping Ji

**Affiliations:** 1 College of Stomatology, Chongqing Medical University, No. 426 Songshibei Road, Chongqing, 401147 China; 2 Chongqing Key Laboratory of Oral Diseases and Biomedical Sciences, Chongqing, China; 3 Chongqing Municipal Key Laboratory of Oral Biomedical Engineering of Higher Education, Chongqing, China

**Keywords:** Validation, TSK-TMD, Temporomandibular disorders

## Abstract

**Background:**

The aim of the study was to validation of the Tampa Scale for Kinesiophobia for Temporomandibular Disorders (TSK-TMD) for use in patients with painful TMD.

**Methods:**

The original TSK-TMD was translated and cross-culturally adaptated following international guidelines. A total of 160 patients with temporomandibular disorders (TMD) were enrolled to fill out the scale. The internal consistency and test-retest methods were used to evaluate the reliability of the TSK-TMD. The validity of the TSK-TMD was analyzed by content validity, construct validity and convergent validity. Construct validity was assessed based on exploratory factor analysis (EFA), and convergent validity by examining the correlation between the global rating of oral health question and TSK-TMD scores.

**Results:**

Cronbach’s alpha value for the total TSK-TMD score was 0.919 and the intraclass correlation coefficient (ICC) value for the TSK-TMD was 0.797. Construct validity was assessed by EFA, extracting two factors, accounting for 71.9% of the variance. The factor loadings of all items were higher than 0.40. In terms of convergent validity, the TSK-TMD subscales showed good correlations to the global rating of oral health question.

**Conclusion:**

These findings show that the Chinese version of TSK-TMD has satisfactory psychometric properties and is appropriate for use in patients with painful TMD in China.

## Background

Temporomandibular disorders (TMD) are now defined as “a group of biopsychosocial illnesses characterized by chronic painful conditions and dysfunction in the muscles of mastication and the temporomandibular joint” [1]. It was reported that 14.9%–17.9% of Chinese people suffered from TMD [2, 3]. The causative factors for the various signs and symptoms of TMD are multifactorial and related to psychological, functional and structural features [4]. One of this oral disorder’s most common clinical manifestations is pain. And this condition may affect areas such as the eyes, throat and ears, frequently leading to headache and neck pain [4]. Additionally, TMD has been shown have an impact on oral health-related quality of life (OHRQoL) [5].

The term “kinesiophobia” refers to “an excessive, irrational, and debilitating fear of movement and activity resulting from a feeling of vulnerability to painful injury or re-injury” [6]. Recent studies support that kinesiophobia is a predictor of disability and craniofacial pain in patients with TMD [7, 8]. Thus, it has important clinical significance of evaluating and treating of TMD patients [7]. Therefore, there is a need to yield an appropriate tool for the evaluation of fear of movement in these patients.

A 17-item scale, named the Tampa Scale for Kinesiophobia (TSK), was then first introduced by Kori et al. to assess kinesiophobia [6]. The psychometric properties of the scale have been checked in different languages [9–13] and for different pain disorders (e.g., low back pain [14, 15], neck pain [13], Parkinson's disease [16] and heart failure [17]). The Tampa Scale for Kinesiophobia for Temporomandibular Disorders (TSK-TMD) was recently developed by Visscher et al. to assess of fear of movement in TMD patients [18]. It was confirmed that the 12-item, two-factor solution of TSK-TMD had generally good reliability and validity.

In recent years, there has been growing interest in applying existing measures for use in different languages and cultures. Mandarin Chinese is one of the most widely spoken languages; however, a Chinese version of TSK-TMD has not been available until now. Therefore, the aims of this study were to develop an appropriate Chinese version of TSK-TMD and to describe the psychometric properties of the Chinese version of TSK-TMD for use in patients with painful TMD.

## Methods

### Patients

A cohort of 160 consecutive patients with a diagnosis of TMD was enrolled from the Stomatological Hospital of Chongqing Medical University between October 2014 and August 2015. The Research Diagnostic Criteria for Temporomandibular Disorders (RDC/TMD-Axis I) was adopted in the present study [19]. The inclusion criteria were as follows: age ≥18 years, ability to understand the TMD questions, and have been symptomatic for painful TMD at least 6 months. Patients who have tooth pain, a systemic disease, a history of psychiatric illness, and were unwilling to consent were excluded.

For the sample size, it was calculated based on the quality criteria for health status questionnaires [20]. The criteria suggested 5–10 patients per question for the measure assessment [20]. Thus, a minimum of 60 patients were required because the TSK-TMD included 12 items. Before the patients filled out the TSK-TMD, a detailed explanation for the content was provided. At the beginning of the first dental treatment, patients were asked to fill out the scale by themselves inside a waiting room. If they cannot fully understand content of the questions, they can consult research assistants at any time.

The Ethics Committee of Chongqing Medical University approved the present study, and every patient signed a written informed consent.

### The TSK-TMD

The original TSK-TMD is an English-language instrument, developed by Visscher et al. in the Academic Centre for Dentistry Amsterdam (ACTA), The Netherlands [18]. It contains 12 items divided into two domains to assess fear of movement in patients with TMD. The response is a four-point Likert scale: strongly disagree, somewhat disagree, somewhat agree, and strongly agree (equivalent to scores of 1–4). High and low total scores of TSK-TMD indicate high and low degrees of kinesiophobia, respectively. Additionally, an extra global question ("*In general, how would you rate your temporomandibular joint status*") was added at the end of questionnaire to evaluate the convergent validity. The statements to the question were: very good, good, fair, poor, very poor, and scores of 1–5, respectively.

### Translation and cross-cultural adaptation

According to standard guidelines proposed by Guillemin et al. [21], the process of translation and cross-cultural adaptation of the TSK-TMD included following major steps:

1. Two independent researchers first translated the TSK-TMD from English to Chinese. The two researchers were fluent in Chinese and English, and also had background knowledge of TMD.
2. Then, the two independent translations were back-translated from Chinese to English by an English teacher without a medical background and two bilingual dental specialists. All of them did not know the original TSK-TMD.
3. An expert panel then compared and assessed the two versions. The panel comprised of two dental experts, two public health experts familiar with health status measures who were fluent in both Chinese and English. An initial Chinese version of TSK-TMD was then produced.
4. The initial Chinese version of TSK-TMD was pilot evaluated in a cohort of 20 patients with painful TMD.
5. After the test, new emerging issues were analyzed and discussed. For example, it was found that the item ‘something potentially harmful going on’ was difficult to understand. We added a brief explanatory note “your own opinions about causes of TMD” at the end of the item to help patients fully understand the meaning. The expert panel considered all of findings and then produced the final Chinese TSK-TMD.

### Statistical analysis

Validity: The content validity, construct validity and convergent validity were analyzed to assess validity of the Chinese version of TSK-TMD.

Content validity refers to the relevance and comprehensiveness of a measure's items.

The expert panel were required to verify whether the Chinese version of TSK-TMD clearly described the measure's purpose, the concepts being measured and the target populations [20]. In the meanwhile, patients were interviewed to determine whether they had difficulties in understanding the items in the pilot trial.

Construct validity was assessed using exploratory factor analysis (EFA). However, the Kaiser–Meyer–Olkin (KMO) test and Bartlett’s test must be firstly carried out to determine whether there are sufficient significant correlations among questions to perform the analysis [22]. Varimax rotation method was adopted in the EFA, and factor loadings higher than 0.40 were considered to be significant.

Finally, convergent validity was assessed by examining the correlation between TSK-TMD total scores and the global oral health question. At the same time, the correlation values is considered to indicate good correlation when 0.41–0.60, to signify very good correlation when 0.61–0.80, and to signify excellent correlation when > 0.81 [23].

Reliability: The criterion of Cronbach’s alpha > 0.70 and the Intraclass Correlation Coefficients (ICC) > 0.70 were used to assess the internal reliability and test-retest reliability, respectively [20]. The value of ICC was calculated using data from 30 patients who completed the Chinese version of TSK-TMD again after a 2-week interval. There was no treatment during these 2 weeks.

All the statistical analysis were performed using the SPSS version 20.0 (IBM Corp. 2011; NY; USA). The *P* value of <0.05 was considered statistically significant in the present study.

## Results

### Patient characteristics

In total, 160 patients were selected from a university-affiliated dental clinic for the study. All the TSK-TMD items were completed fully and the patients reported that the items were readily understandable. The mean age of the patients was 45.2 ± 15.8 years, and the 54.4% were female. The demographic and characteristics of the patients are presented in Table 1. Table 2 presents the mean scores, corrected item-total correlations and Cronbach’s alpha if item deleted results for the TSK-TMD.

**Table 1:** Characteristics of patients (*n* = 160)

		Value
Age (years)	Mean (SD)	45.2 ± 15.8
Gender (n)	Male	73 (45.6%)
	Female	87 (54.4%)
Employment (n)	Employed	113 (70.6%)
	Unemployed	47 (29.4%)
Education history (n)	Primary School	39 (24.4%)
	Middle school	83 (51.9%)
	Bachelor degree or above	38 (23.7%)
Classification of pain (n)	Joint pain	49 (30.6%)
	Muscle pain	58 (36.3%)
	Mixed pain	53 (33.1%)

**Table 2:** Range, mean scores, corrected item-total correlations and factor analysis results for the TSK-TMD

Item	Mean (SD)	Corrected item-total correlation	Cronbach’s alpha if item deleted	Factor loading	
				Factor 1	Factor 2
1. Injure myself	2.87 (0.74)	0.742	0.908	**0.828**	0.270
2. Get worse	2.63 (0.65)	0.698	0.910	**0.726**	0.314
3. Something is seriously wrong	2.34 (0.98)	0.779	0.908	0.389	**0.809**
4. Not take my jaw symptoms seriously	1.98 (0.59)	0.749	0.909	0.440	**0.686**
5. Put my health at risk	2.71 (0.76)	0.423	0.918	-0.147	**0.931**
6. Have injured my jaw	2.23 (0.59)	0.778	0.908	0.285	**0.890**
7. Not to move my jaw	2.59 (0.68)	0.845	0.904	**0.868**	0.365
8. Something potentially harmful going on	1.99 (0.64)	0.674	0.911	0.358	**0.693**
9. Know when to stop moving jaw	2.63 (0.66)	0.828	0.905	**0.850**	0.362
10. Cannot do everything	2.19 (0.65)	0.382	0.918	**0.804**	-0.194
11. No one should have to move jaw	2.31 (0.49)	0.574	0.916	**0.656**	0.235
12. Afraid to open mouth	2.62 (0.66)	0.633	0.913	**0.558**	0.398

The bold values refer to the factor loadings of items. Factor loadings >0.40 were considered significant in the present study

### Validity

Overall, the content validity of the TSK-TMD was considered adequate in the pilot trial. The expert panel concluded independently that the concepts being investigated were clearly defined, and all items were estimated relevant for assessing painful TMD in China. In the meantime, the patients did not mention any unreadable questions.

The result of the KMO test was 0.705 and Bartlett's test of sphericity was 2169.9 (df = 66, *P* < 0.001). Based on these results, it could be demonstrated that there were sufficient significant correlations to perform EFA. Table 2 presents the results of the EFA for the TSK-TMD. All items had factor loadings above 0.40. Two factors were established which together accounted for 71.9% of the total variance. In the EFA, the first factor comprised questions related to ‘activity avoidance’ (items 1, 2, 7, 9–12). It reflects the belief that activity may cause injury or increased pain. The second factor mainly addressed the belief in underlying and serious medical problems, which was labelled ‘somatic focus’ (items 3–6, 8).

Finally, the convergent validity was analyzed by the global rating of oral health question and the Pearson Correlation Coefficient. The results showed good correlations (*r*_*s*_ = 0.458–0.563).

### Reliability

Cronbach’s alpha for the whole score of TSK-TMD was 0.919 and values of the subscales ranged from 0.895 for ‘somatic focus’ to 0.907 for ‘activity avoidance’ (Table 3). The ICC was 0.797 for the total scale and ranged from 0.760 to 0.807 for the subscales of the TSK-TMD (Table 3).

**Table 3:** Internal consistency, test–retest reliability and convergent validity of the TSK-TMD

Subscale	Corrected item-total correlation (*n* = 160)	Test–retest (ICC, 95% CI) (*n* = 30)	*r*_*s*_^a^ (95% CI) (*n* = 160)
Total score	0.919	0.797 (0.595-0.939)	0.563(0.414-0.703) **
Subscales			
Activity avoidance	0.907	0.807 (0.574-0.956)	0.458(0.319-0.592) **
Somatic focus	0.895	0.760 (0.552-0.934)	0.471 (0.308-0.615) **

*ICC* Intraclass correlation coefficient

^a^ Spearman’s rank correlation coefficient

** *P* < 0.001

## Discussion

To the best of our knowledge, this study provides the first introduction and evaluation of the psychometric properties of the TSK-TMD in a non-English-speaking country. The results suggested that the 12-item Chinese version of TSK-TMD with two factors has adequate internal reliability, test-retest reliability, content validity, construct validity and convergent validity.

In the present study, the Chinese version of TSK-TMD was produced in accordance with international guidelines [21]. The procedure contained a process of forward and back translation, conceptual equivalence confirmation by a convenience sample of patients and an expert panel. The results proved that linguistic and cultural equivalence of English and Chinese versions of TSK-TMD.

EFA is one of the most commonly used statistical methods to establish the dimensionality of instruments. The EFA results suggest that the Chinese version of TSK-TMD can be best characterized by two factors as originally proposed by Visscher et al. [18]. The first domain, labelled ‘activity avoidance’, includes 7 items (items 1, 2, 7, 9–12). It seems that those questions are frequently observed reactions in TMD patients, especially in those who try to avoid their jaw from making sounds on movements [18]. Another factor, ‘somatic focus’, comprises questions represents the TMD patient’s belief that the complaints are related to a serious medical problem [18]. It is closely associated with catastrophizing thoughts. In the convergent validity test, significant correlations were observed between TSK-TMD scores and global oral health rating. Overall, the Chinese TSK-TMD demonstrated good convergent validity.

Cronbach’s alpha for the overall TSK-TMD was 0.919, higher than the original study in the Netherlands [18]. The corrected item-total correlations for the 12 items were all higher than the minimum recommended level of 0.2 [24]. In the meanwhile, there was no need to delete any item from the TSK-TMD, because the whole alpha value did not increase when any item was deleted. The test-retest reliability for the overall TSK-TMD was regarded as good with an ICC value of 0.797 that was very near to 0.8 and thus exhibited good reproducibility.

However, some limitations in the present study should be noted. Firstly, all patients were recruited from a university-affiliated clinic. It is essential to replicate of the findings in a general population. Furthermore, a longitudinal study is required to estimate the sensitivity and responsiveness of the Chinese version of TSK-TMD.

## Conclusion

The TSK-TMD was linguistically translated into Chinese and culturally adapted for use in China. Meanwhile, internal reliability, test-retest reliability, content validity, construct validity and convergent validity of the measurement have been confirmed in a sample of TMD patients. The results provide initial evidence that the Chinese TSK-TMD is appropriate for clinical uses in China.

## Abbreviations

EFA: Exploratory factor analysis
ICC: Intraclass correlation coefficient
KMO: Kaiser–Meyer–Olkin test
OHRQoL: Oral health-related quality of life
RDC/TMD: Research diagnostic criteria for temporomandibular disorders
TMD: Temporomandibular disorders
TSK-TMD: The Tampa Scale for Kinesiophobia for Temporomandibular Disorders
